# The complete mitochondrial genome of the Thick-billed Flowerpecker (*Dicaeum agile*)

**DOI:** 10.1080/23802359.2021.1927872

**Published:** 2021-05-19

**Authors:** Yong Gao, Si Yin, Lei Zhu

**Affiliations:** College of Biological Resource and Food Engineering, Qujing Normal University, Qujing, China

**Keywords:** Thick-billed Flowerpecker, Dicaeum agile, mitochondrial genome, phylogenetic analysis

## Abstract

The Thick-billed Flowerpecker (*Dicaeum agile*), a small member of the flowerpecker family, inhabits tropical Southern Asia, and is widely distributed from India east to Indonesia and Timor. In this study, next generation sequencing (NGS) was used to obtain a mitochondrial genome sequence for *D. agile*. The complete mitogenome was 16,809bp in length, with a GC content of 46.40%. The genome sequence contained thirteen protein-coding genes, one 12S RNA gene, one 16S RNA gene, and 22 tRNA genes. A phylogenetic tree constructed for the family confirmed that *D. agile* is closely related to another species of *Dicaeum* (*Dicaeum eximium*). The mitochondrial genome of the Thick-billed Flowerpecker will be useful for future phylogeographic studies of this species.

The Thick-billed Flowerpecker (*Dicaeum agile*) is a tiny bird in the flowerpecker family, feeding predominantly on fruits. Thick-billed Flowerpeckers are active birds that are mainly seen in forest treetops. Members of this species are about 10 cm long with a dark stout beak and short tail (Cheke and Mann 2008). Dullish gray in color, the birds are darker gray brown on top, and have diffuse streaking on their light buff underparts. The bill is dark, somewhat stout and heavy, and the iris is reddish (Sheldon 1985). A resident of tropical Southern Asia, the species is widely distributed from India east to Indonesia and Timor; several populations are recognized as subspecies (Nyári et al. 2009).

To provide genomic resources for studying genetic diversity and structure in *D. agile*, next generation sequencing (NGS) was used to obtain a mitochondrial genome sequence for this species. A sample was collected from Mengla (E 101°32′28″, N 21°27′34.2″), in Yunnan Province, China, in August 2020, and the specimen was stored in the herbarium of College of Biological Resource and Food Engineering, Qujing Normal University (voucher no. QJNU-Zhu-20200804). Total genomic DNA was extracted from a blood sample using a commercial blood DNA isolation kit (DP318; TIANGEN, Beijing, China). A DNA sequencing library was then prepared and paired-end sequenced with a read length of 150 bp (PE150) on an Illumina Novaseq platform (Illumina, San Diego, CA). A total of 5.35 Gb genomic data (17,854,552 reads) was produced, and mtDNA reads were identified by aligning genomic sequences against the mitochondrial genome of *Dicaeum eximium*. Identified sequences were assembled using SPAdes version 3.5.0 (http://cab.spbu.ru/software/spades/) (Bankevich et al. 2012). Protein-coding genes were analyzed using ORF Finder (http://www.ncbi.nlm.nih.gov/gorf/gorf.html), while tRNA and rRNA genes were identified using tRNA-scan SE version 1.3.1 (Lowe and Eddy 1997) and RNAmmer version 1.2 (Lagesen et al. 2007), respectively. To infer the phylogenetic position of *D. agile*, mitogenomes of twelve birds were downloaded from NCBI (Hsieh et al. 2018; Wang et al. 2019; Feng et al. 2020; Yang et al. 2020). The *D. agile* mitogenome was aligned to other mitogenomes using MUSCLE (Edgar 2004). A maximum-likelihood (ML) tree was constructed in MEGA 7 (Kumar et al. 2016) using the GTR + gamma model and 1000 bootstrap replicates. Lastly, the phylogenetic tree was visualized in Figtree version 1.4.3 (http://tree.bio.ed.ac.uk/software/figtree/).

The complete circular mitogenome of *D. agile* (NCBI GenBank accession no. MW429431) was 16,809bp in length with a GC content of 46.40%. The base composition was 30.13% A, 31.45% C, 14.95% G, and 23.47% T. The genome contained one 12S RNA gene, one 16S RNA gene, and 22 tRNA genes. Thirteen protein-coding genes were detected across the whole mitogenome, and the average gene length was 900 bp. The phylogenetic analysis confirmed that *D. agile* was sister to another *Dicaeum* species (*D. eximium*). A relatively close relationship with *Dicaeum* and a member of the genus *Leptocoma* was also suggested by the ML tree (Figure 1).

**Figure 1.**
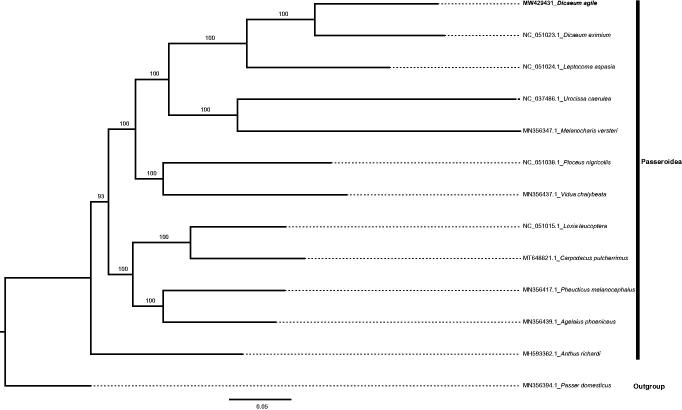
The maximum-likelihood tree constructed using mitochondrial genome sequences from *Dicaeum agile* and eleven other Passeroidea species (bootstrap values based on 1000 replicates).

## Data Availability

The genome sequence data that support the findings of this study are openly available in GenBank of NCBI at (https://www.ncbi.nlm.nih.gov/) under the accession no. MW429431. The associated BioProject, SRA, and Bio-Sample numbers are PRJNA706876, SRR13862217, and SAMN18147071 respectively.
